# Addressing NCDs: Challenges From Industry Market Promotion and Interferences

**DOI:** 10.15171/ijhpm.2019.02

**Published:** 2019-01-20

**Authors:** Viroj Tangcharoensathien, Orana Chandrasiri, Watinee Kunpeuk, Kamolphat Markchang, Nattanicha Pangkariya

**Affiliations:** International Health Policy Program, Ministry of Public Health, Nonthaburi, Thailand.

**Keywords:** Non-Communicable Diseases, Industry Interference, Best Buy NCD Interventions, Regulatory Capture, NCD Risk Factors

## Abstract

Addressing the determinants of non-communicable diseases (NCDs) is challenged by aggressive market promotion by tobacco, alcohol and unhealthy food industries in emerging countries with fast economic development; and interference by these industries in government policies aimed at containing consumption of unhealthy products. This editorial reviews market promotion and industry interference and classifies them into four groups of tactics: (a) interfering with the legislative process; (b) using front groups to act on their behalf; (c) questioning the evidence of tobacco harm and the effectiveness of harm-reduction interventions; and (d) appearing responsible in the eyes of the public, journalists and policy-makers. Despite active implementation of the Framework Convention on Tobacco Control (FCTC), the tobacco, alcohol and unhealthy food industries use similar tactics to aggressively interfere in policies, with the tobacco industry being the most aggressive. Policy interference by industries are effective in the context of poor governance, rampant corruption, conflict of interest among political and government actors, and regulatory capture in all levels of countries from low- to high-income. In addressing these interferences, government requires the practice of good governance, effective mechanisms to counteract conflict of interests among political and policy actors, and prevention of regulatory capture. The World Health Organization (WHO) Framework of Engagement with non-State Actors can be applied to the country context when engaging private entities in the prevention and control of NCDs.

## Introduction

In the context of trade liberalization, which promotes the availability and consumption of goods and services through reduction of taxes and tariffs, the increased consumption of alcohol, unhealthy foods, and breast milk substitutes (BMS) is boosted by aggressive market promotion. This is particularly so in countries with fast economic growth and increasing household disposal income. Governments’ efforts to prevent and control non-communicable diseases (NCDs) through increased tax and price, and controlled advertising of these products are hampered by policy interference by alcohol, tobacco, and unhealthy food industries.^1^

In response to the complex intersection between trade and health, the World Health Assembly Resolution WHA59.26 on International Trade and Health^2^ urges Member States to convene multi-stakeholder dialogue using a national platform for harmonizing sectoral policies and preventing negative trade impacts on the health of the population. Progress has been slow as it was limited by the lack of capacity in the health sector to convene partnerships with trade and commerce, to generate evidence for policy, and to track the implications of trade agreements relating to the health of the population.^3^ Furthermore, government policies to contain NCDs such as taxes, labeling with health warnings, and restricted advertising and marketing are often interfered by industries; this hampers the primary prevention of NCDs.

Alcohol use was the seventh leading risk factor for 2.2% (95% uncertainty interval [UI] 1.5–3.0) of age-standardized female deaths and 6.8% (5.8–8.0) of age standardized male deaths in 2016. Mortality impact is much higher among prime adults (15-49 years old). The study concludes that the level of alcohol consumption that minimizes harm across health outcomes is zero standard drinks per week.^4^

To tackle the determinants of NCDs, the World Health Organization (WHO) suggests some ‘best buy’ interventions. These are defined through an average cost-effectiveness ratio of international dollars ≤100 per disability-adjusted life years averted in low- and lower middle-income countries.^5^ A systematic review shows that tobacco warnings increase people’s attention to and recall of health messages, which ultimately reduces smoking and motivates quitting.^6^ Graphic pictures enhance the effectiveness of warning labels.^7^ A randomized control trial among Dutch children shows that sugar sweetened beverage (SSB) intake affects relative weight.^8^ In a systematic review, the own-price elasticity for soft drinks is -1.30 (95% CI: -1.09 to -1.51)^9^; which indicates that for every one percent increase in SSB price, its demand drops by 1.3%. Evidence shows that 20% SSB tax in Australia is likely to decrease SSB purchase and consumption, leading to significant health gains and healthcare expenditure savings.^10^ In California,^11^ SSB taxes are effective in shifting consumers to purchase healthier beverages without causing undue economic hardship, while raising revenue for social objectives. One-cent-per-ounce excise tax on SSB implemented across the United States would have prevented nearly 580 000 cases of childhood obesity and more than US$30 in health care cost savings for every US$1 investment.^12^

This editorial reviews evidence on market promotion and policy interference by tobacco, alcohol and food industries when governments introduce measures to contain consumption of these products in order to prevent and control NCDs; and suggests how governments might find ways to overcome these challenges.

## Emerging Countries: Targets of Market Promotion and Health Implications

Emerging countries in Asia had the highest gross domestic product (GDP) growth rate between 2008 and 2017 at an average of 7.2%; more than double the world’s 3.2% average (see Table). The European Union had a 0.7% ten-year average growth. Emerging countries are therefore the best targets for market promotion of tobacco, alcohol, BMS and SSB.

**Table:** World’s GDP Growth by Region Between 2008 and 2017 by Percent

**Country Group Name**	**2008**	**2009**	**2010**	**2011**	**2012**	**2013**	**2014**	**2015**	**2016**	**2017**	**Average Growth 2008-2017**
Emerging and developing Asia	7.2	7.5	9.6	7.8	6.9	6.9	6.8	6.6	6.4	6.3	7.2
ASEAN^a^	5.4	2.4	6.9	4.7	6.2	5.1	4.6	4.8	4.8	5.1	5
Emerging market and developing economies	5.8	3	7.4	6.3	5.3	4.9	4.6	4	4.1	4.6	5
Sub-Saharan Africa	6	4	6.6	5	4.3	5.2	5.1	3.4	3	4	4.7
World	3	-0.1	5.4	4.2	3.5	3.3	3.4	3.1	3.2	3.5	3.2
Emerging and developing Europe	3.1	-3	4.7	5.4	1.2	2.8	2.8	3.5	3.5	3.3	2.7
Advanced economies	0.2	-3.4	3.1	1.7	1.2	1.2	1.8	1.9	1.9	2	1.1
European Union	0.7	-4.3	2	1.8	-0.4	0.3	1.4	2	1.8	1.9	0.7

Abbreviation: GDP, gross domestic product. ^a^ Including Indonesia, Malaysia, Philippines, Vietnam, and Thailand Source: Obiols M. 2017 World’s GDP Growth by Region 2017. https://bit.ly/2PW3CFs. Accessed November 17, 2017.

Worldwide, more than one-fifth of the adult population smokes, consuming 5500 billion cigarettes in 2017. Excluding China which has 43% of global market share, the global tobacco market in 2017 was worth US$760 billion. More than US$680 billion was from sales of conventional cigarettes and the remaining market of “Next Generation Products” was predicted to double between 2016 and 2021.^13,14^

The value of the global alcoholic beverages market was US$1439 billion in 2017, and is expected to reach US$1684 billion by 2025. The compound annual growth rate was 2% between 2018 and 2025.^15^ The alcohol market is driven by increased growth in the middle classes and those with disposable incomes, and demand for premium product brands. Flavored alcoholic beverages have also boosted market growth.^16^

Asia is the fastest-growing alcohol market, with more than 30% of global sales in 2014, with a growth of 176% between 2000 and 2019.^17^ Active market promotion in Asia results in significant increases in alcohol consumption among youth.^18^

A systematic review demonstrates a positive association between level of exposure to alcohol marketing and initiation, consumption, and binge consumption among youth. Market promotion increases youth’s brand recognition and receptivity to alcohol.^19^

The global market size of carbonated soft drinks was US$392.6 billion in 2016, boosted by increased consumer disposable income and extensive distribution channels.^20^ A greater consumption of SSB is associated with weight gain and obesity.^21,22^ In Asia, young and middle to upper-income women in major cities are also increasingly purchasing and drinking alcoholic drinks.^23^

In the United kingdom, top food companies spent more than £143 million on product advertising in 2016. The advertising spending was 27.5 times the £5.2 million that the Government spent on a healthy eating campaign, such as ‘Change for life.’^24^

The global market of BMS grew by 40.8% between 2008 and 2013, mostly in East Asian emerging market such as China, Indonesia, Thailand, and Vietnam. It grew from 5.5 to 7.8 kg BMS per infant or child per year; and was predicted to increase to 10.8 kg BMS by 2018. The aggressive marketing of BMS contributes to sub-optimal breastfeeding and adverse effects on child and maternal health outcomes.^25^ Not providing breastfeeding is associated with lower intelligence, delayed cognitive development and economic losses of about US$302 billion annually or 0.49% of world gross national income.^26^ Exclusive breastfeeding of newborns for 6 months in China went down steadily from 67% in 1998 to 50.8% in 2003, 27.6% in 2008 and 18.6% in 2013.

## Industry Interferences

When governments introduce tough measures against NCD risk factors, the tobacco,^27-29^ alcohol,^30^ food and beverage industries^31^ – including their proxies – interfere with public policy development.

Article 5.3 of the Framework Convention on Tobacco Control (FCTC) states: “In setting and implementing their public health policies with respect to tobacco control, Parties shall act to protect these policies from commercial and other vested interests of the tobacco industry in accordance with national law.” This serves as a foundation for State Parties to keep vigilance and safeguard themselves from undue influences by tobacco industry. This good practice can be applied to other harmful products.

### Tobacco Industry

The tobacco industry applies legal threats and files law suits against governments.^32-35^ In emerging African markets, their petition to Kenya’s high court against the Government’s proposed tobacco regulations on April 16, 2015 was critical, saying, “Kenya’s proposal for a new 2% tax on tobacco products is arbitrary and capricious; it will have a significant effect on cigarette manufacturers and importers putting at risk further investment, direct and indirect employment opportunities in Kenya.” In Uganda, the tobacco industry’s petition in the constitutional court against the Tobacco Control Act commented the following: “The Tobacco Control Act, read as a whole, has the effect of unjustifiably singling out the tobacco industry for discriminative treatment.”

To counteract a government’s ban on point-of-sales advertising, the industry uses a few counteracting messages. For example, it costs retailers a significant amount to remodel their store or face lost sales as customers buy tobacco elsewhere. Experiences show such bans do not work; it simply does not reduce smoking and bans on point-of-sale display are anti-competitive. Tobacco representatives offer retailers financial, ‘loyalty’ or ‘reward’ schemes^36^ to boost sales volume.

To counteract government’s increased size of health warning label, the tobacco industry applies several tactics. The 30% of package areas are sufficient to fulfill FCTC minimum requirements. The oversized, shocking warning does not reduce smoking rates.^37^ The example of Sweden raised by the industry provides a half-truth; packages apply a text warning only but there is only a very low smoking rate at 13%. The distorted message does not capture other strong control measures in Sweden. Spain applies a graphic warning but the smoking rate is 33%.

The tobacco industry infiltration into the sessions of Conference of Parties (COP) of the FCTC has led to the tighter screening of members of the public and amendments of the Rule of Procedure of the COP to allow only accredited, properly vetted representatives of the media to attend the ‘open’ COP discussions and clarify which of the subsidiary body meetings are restricted or public.^38^

The tobacco industry has interpreted that Article 6 on the guidelines on price and tax measures to reduce the demand for tobacco adopted by COP 6 (2014)^39^ to be a non-legally binding instrument. They see it as the sovereign right of State Parties to determine their tobacco tax policies, where affordability is a key consideration in setting tobacco tax.

### Alcohol Industry

The alcohol industry interfered with the National Institute of Health (NIH)-initiated Moderate Alcohol and Cardiovascular Health trial by providing funding support without due process. This multi-centre randomized clinical trial aimed to determine the effects of one alcohol serving daily on cardiovascular and diabetes outcomes compared with a no alcohol intake group. Industry funding to the Moderate Alcohol and Cardiovascular Health study cast doubt over the scientific integrity and the trial’s independence from industry influence. This irregularity led to the NIH’s decision to terminate the study.^40^

The alcohol industry is highly effective and well organized in gaining access to the policy making process including public consultations, parliamentary committees and working groups. They build close relationships with policy actors such as ministers, political and technical advisors, civil servants officers, members of parliaments, and other political representatives. These approaches minimize threats to their commercial interests.^41^

The alcohol industry casts doubt on a wealth of scientific evidence, promoting weak survey-based evidence, and making unsubstantiated claims to their advantage.^42^ They invest in research on issues where they have clear vested interests in the outcomes. A systematic review identified serious concerns about the inherent conflict of interest between the commercial goals of industry actors and the production and dissemination of public health research.^43^ The International Center for Alcohol Policies, supported by the industry, not only counters WHO recommendations and refutes evidence on the most effective strategies to prevent alcohol-related harm; its recommended alcohol policies are congenial to industry interests.^44^

### Unhealthy Food Industry

When Thailand introduced SSB tax^45^ in 2017 to address obesity, the Government faced serious resistance from the soft drink industry. The Thai Beverage Industry Association questioned the link between obesity and drinking sweetened beverages.^46^

In the United States, the beverage industry pressured California lawmakers, where there are SSB taxes in four cities, for a 12-year moratorium on local SSB taxes. Lawmakers were held “hostage” by the beverage industry, which spent $7 million on a ballot initiative and campaign which would have made it much harder for cities to raise taxes from any sources. The beverage industry dropped the initiative after lawmakers agreed to their proposed SSB tax moratorium.^47^ At the meeting of the Academy of Nutrition and Dietetics, the two largest exhibits belonged to PepsiCo and Ocean Spray, both of which sell sweetened drinks. The Academy still casts doubt on evidence that SSB tax improves health.

## Conclusion and Recommendation

This review shows that tactics used by the alcohol and unhealthy food industries^48^ are similar to the four most common approaches used by the tobacco industry.^49^ All interfere with the legislative process to prevent the adoption of control measures; use front groups to act on their behalf; question the evidence of their product harm and the effectiveness of interventions; and aim to appear responsible in the eyes of the public, journalists and policy-makers. Trade agreements provide platforms for industries to introduce legal challenges and undermine government measures to control tobacco and alcohol, for example, trademark protections and other technical barriers to trade.^50^

These tactics can be especially effective in the context of poor governance, rampant corruption, conflict of interests among political and government actors, as well as regulatory captures.^51^ Similar interference is also reported from high-income countries^52,53^ where there are more capacities and resources to counteract.

Not only good governance, but effective mechanisms to counteract conflict of interests among political and government actors, and prevent regulatory capture are also needed.^54,55^ The WHO Framework of Engagement with non-State Actors^56^ can be applied to country contexts when engaging with private entities in the prevention and control of NCDs. Strengthening the capacities of government lawyers to defend lawsuits by industries and take litigating against law violations is also critical.^57^

## Ethical issues

Not applicable.

## Competing interests

Authors declare that they have no competing interests.

## Authors’ contributions

All authors involved in the conceptualize the paper, design, and analysis. OC, WK, KM, and NP searched and reviewed literature. All authors read and approved the final manuscript.
